# Targeted enrichment outperforms other enrichment techniques and enables more multi-species RNA-Seq analyses

**DOI:** 10.1038/s41598-018-31420-7

**Published:** 2018-09-06

**Authors:** Matthew Chung, Laura Teigen, Hong Liu, Silvia Libro, Amol Shetty, Nikhil Kumar, Xuechu Zhao, Robin E. Bromley, Luke J. Tallon, Lisa Sadzewicz, Claire M. Fraser, David A. Rasko, Scott G. Filler, Jeremy M. Foster, Michelle L. Michalski, Vincent M. Bruno, Julie C. Dunning Hotopp

**Affiliations:** 1 Institute for Genome Sciences, University of Maryland School of Medicine, Baltimore, MD 21201 USA; 2 Department of Microbiology and Immunology, University of Maryland School of Medicine, Baltimore, MD 21201 USA; 3 University of Wisconsin Oshkosh, Oshkosh, WI 54901 USA; 4 Division of Infectious Diseases, Los Angeles Biomedical Research Institute at Harbor-UCLA Medical Center, Torrance, California 90502 USA; 5 New England Biolabs, Ipswich, MA 01938 USA; 6 Department of Medicine, University of Maryland School of Medicine, Baltimore, MD 21201 USA; 7 David Geffen School of Medicine at UCLA, Los Angeles, California, 90502 USA; 8 Greenebaum Cancer Center, University of Maryland, Baltimore, MD 21201 USA

## Abstract

Enrichment methodologies enable the analysis of minor members in multi-species transcriptomic data. We compared the standard enrichment of bacterial and eukaryotic mRNA to a targeted enrichment using an Agilent SureSelect (AgSS) capture for *Brugia malayi*, *Aspergillus fumigatus*, and the *Wolbachia* endosymbiont of *B. malayi* (*w*Bm). Without introducing significant systematic bias, the AgSS quantitatively enriched samples, resulting in more reads mapping to the target organism. The AgSS-enriched libraries consistently had a positive linear correlation with their unenriched counterparts (r^2^ = 0.559–0.867). Up to a 2,242-fold enrichment of RNA from the target organism was obtained following a power law (r^2^ = 0.90), with the greatest fold enrichment achieved in samples with the largest ratio difference between the major and minor members. While using a single total library for prokaryote and eukaryote enrichment from a single RNA sample could be beneficial for samples where RNA is limiting, we observed a decrease in reads mapping to protein coding genes and an increase in multi-mapping reads to rRNAs in AgSS enrichments from eukaryotic total RNA libraries compared to eukaryotic poly(A)-enriched libraries. Our results support a recommendation of using AgSS targeted enrichment on poly(A)-enriched libraries for eukaryotic captures, and total RNA libraries for prokaryotic captures, to increase the robustness of multi-species transcriptomic studies.

## Introduction

Dual species transcriptomic experiments have been increasingly employed as a method to analyze the transcriptomes of multiple species within a system^1–9^. However, obtaining a sufficient quantity of reads from each organism is a major issue when simultaneously analyzing the transcriptomes of multiple organisms within a single sample. When extracting total RNA from a host sample, the signal from host transcripts typically overwhelms the signal from secondary organism transcripts under most biologically meaningful conditions. Differential enrichments have been designed to physically extract RNA from secondary organisms in samples by depleting highly abundant rRNAs and selecting transcripts based on differing properties between the two organisms, such as the differential poly-adenylation status of transcripts in the case of samples containing a mixture of eukaryotic and prokaryotic RNA^10^. However, in eukaryote-eukaryote dual species transcriptomics experiments, these differences are usually not present to be exploited. Additionally, methods used to enrich for prokaryotic RNA from samples dominated by eukaryotic RNA can fail when the poly(A)-depletion method is unable to remove eukaryotic long non-coding RNAs that lack 3′-polyadenylatation, and when the efficacy of rRNA depletion is low, as observed with some organisms.

The Agilent SureSelect (AgSS) platform serves as a hybridization-based enrichment method that uses specific baits to target transcripts of interest (www.genomics.agilent.com). By designing sequence-specific baits for an organism, it becomes possible to extract transcripts of interest, such as mRNA, while avoiding unwanted RNA types, such as rRNA and tRNA. In dual species transcriptomics, one of the main advantages of the AgSS system is the ability to extract reads in systems where an organism has low relative abundance^11^. To test the efficacy of the AgSS platform on dual- and tripartite-species systems where insufficient numbers of reads are obtained from the minor member(s), we designed AgSS baits for: (1) *Brugia malayi*, a filarial nematode and the causative agent of lymphatic filariasis, (2) *w*Bm, the obligate mutualistic *Wolbachia* endosymbiont of *B. malayi*, and (3) *Aspergillus fumigatus* AF293, a fungal pathogen known to cause aspergillosis in immunocompromised individuals.

In filarial nematode transcriptomics, it is relatively straightforward to isolate nematode samples from the mammalian/definitive host while minimizing contaminating host material. Therefore, the *Brugia* transcriptome can be easily obtained from poly(A)-enriched RNA from *Brugia* samples originating from the mammalian host, which is often an experimentally infected gerbil (*Meriones unguiculatus*). However, sampling the *Wolbachia* endosymbiont transcriptome in these worms is problematic because the endosymbionts contribute less RNA to the total RNA pool. Therefore, *w*Bm reads are overwhelmed by *B. malayi* reads and must be enriched using a rRNA- and poly(A)-depletion, which can still result in an insufficient number of reads being obtained. In the mosquito vector host, the parasitic worms develop in the thoracic muscles where they are not easily isolated. Therefore, whole infected mosquito thoraces containing the larval *B. malayi* are used for RNA isolation. As such the *B. malayi* reads are overwhelmed by reads from those of the mosquito vector, typically experimentally infected *Aedes aegypti*, and the reads of the endogenous *w*Bm are even further dwarfed compared to samples obtained from the definitive host. In a separate dual-species transcriptomics experiment examining *A fumigatus* infections, the eukaryotic *A. fumigatus* reads are overwhelmed by reads from the eukaryotic human or mouse host making it difficult to analyze the fungal transcriptome in this host-pathogen interaction. Given that both are eukaryotes there are not differences in characteristics like polyadenylation to enable the enrichment of the mRNA from the minor member, specifically *A. fumigatus*.

For each eukaryotic system, the performance of the poly(A)-enrichment was compared to that of poly(A)-enrichment supplemented with the AgSS platform in extracting *B. malayi* and *A. fumigatus* reads. Additionally, the efficacy of the AgSS platform in extracting prokaryotic reads was determined by comparing the total RNA AgSS capture to the RiboZero-treated, poly(A)-depletion method in extracting *w*Bm reads.

## Methods

### Mosquito preparation

*Aedes aegypti* black-eyed Liverpool strain mosquitoes were obtained from the NIH/NIAID Filariasis Research Reagent Resource Center (FR3) and maintained in the biosafety level 2 insectary at the University of Wisconsin Oshkosh (UWO). Desiccated mosquito eggs were hatched in deoxygenated water and the larvae maintained on a slurry of ground TetraMin fish food (Blacksburg, VA, USA) at 27 °C and 80% relative humidity. Female pupae were separated from males using a commercial larval pupal separator (The John Hock Company, Gainesville, FL, USA) and maintained on cotton pads soaked in sucrose solution. Adult female mosquitos were deprived of sucrose ~8 h prior to blood feeding. Mosquitoes were infected with the *B. malayi* FR3 strain by feeding on microfilaremic cat blood (FR3) through parafilm via a glass jacketed artificial membrane feeder. Microfilaremic cat blood was diluted using uninfected rabbit blood to achieve a suitable parasite density for infection (100–250 mf (microfilariae)/20 µL) with mosquitoes being allowed to feed to repletion. Mosquitoes were maintained in insect incubators until time of worm harvest.

### Nematode preparation

To examine larval development in the vector, groups of mosquitoes were sampled at 18 h post infection (hpi), 4 days post infection (dpi), and 8 dpi. Because larval development occurs in the thoracic muscle of the mosquito, thoraces of infected mosquitoes containing larval *B. malayi* were (1) separated from the head, abdomen, legs, and wings, (2) flash frozen in liquid nitrogen, (3) and stored at −80 °C prior to RNA isolation. To generate third stage larvae (L3) of *B. malayi*, infected mosquitoes at 9–16 dpi were processed in bulk using the NIAID/NIH Filariasis Research Reagent Resource Center (FR3) Research Protocol 8.4 (www.filariasiscenter.org). Larvae were isolated in RPMI media containing 0.4 U penicillin and 4 µg streptomycin per mL (RPMI + P/S), flash frozen in liquid nitrogen, and stored at −80 °C. To generate fourth larval stages (L4) and adult worms, freshly isolated L3s were injected into the peritoneal cavities of Mongolian gerbils (*Meriones unguiculatus*). Briefly, male Mongolian gerbils three months of age or older (Charles River, Wilmington, MA, USA) were anesthetized with 5% isoflurane, immobilized on a thermal support, and administered ocular Paralube. The inguinal region for each gerbil was shaved and disinfected with iodine. Infections were performed by delivering L3 larvae into the peritoneal cavity using a butterfly catheter, which was left in place and flushed afterwards with 1 mL warmed RPMI + P/S to ensure delivery of all larvae. Afterwards, gerbils were removed from the plane of anesthesia and allowed to fully recover in a hospital cage prior to returning to group housing. Worms were later harvested by euthanizing the gerbils and soaking the peritoneal cavities in warm RPMI + P/S. Worms were washed in warm RPMI + P/S to remove traces of gerbil tissue, flash-frozen with liquid nitrogen, and stored at −80 °C. All animal care and use protocols were carried out in accordance with the relevant guidelines and regulations and were approved by the UWO IACUC.

### Mosquito/Nematode/*Wolbachia* RNA isolation

Mosquito thoraces were combined with TRIzol (Zymo Research, Irvine, CA, USA) at a ratio of 1 mL TRIzol per 50–100 mg mosquito tissue while nematode samples were processed using a 3:1 volume ratio of TRIzol to sample. β-mercaptoethanol was added to a final concentration of 0.1%. The tissues were homogenized in a TissueLyser (Qiagen, Germantown, MD) at 50 Hz for 5 min. The homogenate was transferred to a new tube and centrifuged at 12,000 × g for 10 min at 4 °C. After incubating at room temperature for 5 min, 0.2 volumes of chloroform were added. The samples were shaken by hand for 15 s, incubated at room temperature for 3 min, then loaded into a pre-spun, phase lock gel heavy tube (5Prime, Gaithersburg, MD, USA) and centrifuged for 5 min at 12,000 × g at 4 °C. The upper phase was removed to a new tube and one volume of 100% ethanol was added prior to loading onto a PureLink RNA Mini column (Ambion, Austin, TX). The samples were then processed following manufacturer instructions, quantified using a Qubit fluorometer (Qiagen, Germantown, MD, USA) and/or a NanoDrop spectrometer (NanoDrop, Wilmington, DE, USA). The RNA was subsequently treated with the TURBO DNA-*free* kit (Ambion, ThermoFisher Scientific, Waltham, MA, USA) according to the manufacturer’s protocol.

### *A. fumigatus* infection

Two different models of invasive aspergillosis, both using male BALB/c mice, were employed^12,13^. In the non-neutropenic model, the mice were immunosuppressed with 5 doses of cortisone acetate, with 500 mg/kg administered subcutaneously every other day, starting four days pre-infection. In the neutropenic model, the mice were administered cyclophosphamide, 250 mg/kg intraperitoneally, and cortisone acetate, 250 mg/kg subcutaneously, two days pre-infection. Four days post infection, the mice were given a second dose of cyclophosphamide, 200 mg/kg intraperitoneally, and cortisone acetate, 250 mg/kg subcutaneously. All mice were infected by placing them in a chamber containing an aerosol of *A. fumigatus* conidia for 1 h. On days 2 and 4 for the non-neutropenic model and on days 4 and 7 for the neutropenic model, 3 mice per time point were sacrificed, after which their lungs were harvested and stored in RNAlater (Ambion, ThermoFisher Scientific, Waltham, MA, USA) for subsequent RNA isolation.

### *A. fumigatus* RNA isolation

Each lung sample was placed in a lysing matrix C tube (MP Biomedicals, Santa Ana, CA, USA) with a single 0.25 inch diameter ceramic sphere (MP Biomedicals, Santa Ana, CA, USA) and homogenized with a bead beater (FastPrep FP120, Qbiogene, Montreal, Quebec, Canada).The RNA was isolated using the RiboPure kit (Ambion, ThermoFisher Scientific, Waltham, MA, USA) following the manufacturer’s instructions, treated with DNase I (ThermoFisher Scientific, Waltham, MA, USA), and purified using the RNA clean & concentrator kit (Zymo Research, Irvine, CA, USA).

### Illumina NEBNext Ultra Directional RNA libraries without capture

Whole transcriptome libraries were constructed for sequencing on the Illumina platform using the NEBNext Ultra Directional RNA Library Prep Kit (New England Biolabs, Ipswich, MA, USA). When targeting eukaryotic mRNA, polyadenylated RNA was isolated using the NEBNext Poly(A) mRNA magnetic isolation module. When targeting bacterial mRNA, samples underwent rRNA- and poly(A)-reductions, as previously described^10,14^. SPRIselect reagent (Beckman Coulter Genomics, Danvers, MA, USA) was used for cDNA purification between enzymatic reactions and size selection. For indexing, the PCR amplification step was performed with primers containing a 7-nt index sequence. Libraries were evaluated using the GX touch capillary electrophoresis system (Perkin Elmer, Waltham, MA) and sequenced on a HiSeq2500, generating 100-bp paired end reads.

### AgSS probe design

Capture probes were designed using the SureSelect DNA Advanced Design Wizard for *B. malayi*, *A. fumigatus*, and *w*Bm. Probes were designed to capture every 120 bp for each coding sequence in each of the three organisms with no overlap, except at one end to ensure the entire coding sequence was covered. DustMasker was used to identify and mask low complexity regions of the genome from the probe design^15^. A total of 167,997 probes were designed for *B. malayi* to capture 11,085 genes, ranging from 1–540 probes/gene with an average of 15.3 probes/gene. For *A. fumigatus*, 126,394 probes were designed to capture 9,835 genes, ranging from 1–213 probes/gene with an average of 12.9 probes/gene. For *w*Bm, 6,393 probes were designed with 1–72 probes/gene and an average of 8.0 probes/gene. The baits were not tested for specificity against the host genome.

### *Wolbachia* AgSS RNA capture

Pre-capture libraries were constructed from 500–1000 ng of total RNA samples using the NEBNext Ultra Directional RNA Library Prep kit (NEB, Ipswich, MA, USA). First strand cDNA was synthesized without mRNA extraction to retain non-polyadenylated transcripts and was fragmented at 94 °C for 8 min. After adaptor ligation, cDNA fragments were amplified with 10 cycles of PCR before capture. *Wolbachia* transcripts were captured from 200 ng of the amplified libraries using an Agilent SureSelectXT RNA (0.5–2 Mbp) bait library designed specifically for *w*Bm. Library-bait hybridization reactions were incubated at 65 °C for 24 h then bound to MyOne Streptavidin T1 dynabeads (Invitrogen, Carlsbad, CA, USA). After multiple washes, bead-bound captured library fragments were amplified with 18 cycles of PCR. The libraries were loaded on a HiSeq4000 generating 151-bp paired end reads.

### *B. malayi* AgSS RNA capture

Pre-capture libraries were constructed from 1000 ng of total RNA samples using NEBNext Ultra Directional RNA Library Prep kit (NEB #E7420, Ipswich, MA, USA). Except where noted in the text, poly(A)-enrichment according to the manufacturer’s protocol was used. After adaptor ligation, cDNA fragments were amplified for 10 cycles of PCR before capture. *B. malayi* transcripts were captured from 200 ng of the amplified libraries using an Agilent SureSelectXT Custom (12–24 Mbp) bait library designed specifically for *B. malayi*. Library-bait hybridization reactions were incubated at 65 °C for 24 h then bound to MyOne Streptavidin T1 Dynabeads (Invitrogen, Carlsbad, CA). After multiple rounds of washes, bead-bound captured library fragments were amplified with 16 cycles of PCR. The libraries were loaded on a HiSeq4000 generating 151-bp paired end reads. In comparisons describing captures on poly(A)-enriched libraries with total RNA libraries, total RNA libraries were constructed after first strand cDNA was synthesized without mRNA extraction to retain non-polyadenylated transcripts and fragmented at 94 °C for 8 min.

### *A. fumigatus* AgSS capture

Pre-capture libraries were constructed from 700 ng of total RNA samples using NEBNext Ultra Directional RNA Library Prep kit (NEB #E7420, Ipswich, MA, USA) according to the manufacturer’s protocol. Briefly, mRNA was extracted with oligo-d(T) beads and reverse-transcribed into first strand cDNA with random primers. First strand cDNA was fragmented at 94 °C for 8 min. After adaptor ligation, cDNA fragments were amplified in a thermocycler for 10 cycles before capture. *Aspergillus* transcripts were captured from 100 ng of the amplified libraries using an Agilent SureSelectXT Custom (12–24 Mbp) bait library for *A. fumigatus*. Libraries were incubated at 65 °C for 24 h then bound to MyOne Streptavidin T1 Dynabeads (Invitrogen, Carlsbad, CA, USA). After multiple rounds of washes, bead-bound captured library fragments were amplified with 16 cycles of PCR. The libraries were loaded on a HiSeq4000 generating 151-bp paired end reads.

### Sequence alignment, feature counts, and fold enrichment calculations

The sequence reads originating from all samples used in all *B. malayi* and *A. fumigatus* AF293 comparisons were mapped to the *B. malayi* genome WS259 (www.wormbase.org) or *A. fumigatus* AF293 genome CADRE34 (www.aspergillusgenome.org), respectively, using the TopHat v1.4 aligner^16^. Read counts for all genes were obtained using HTSeq (v0.5.3p9)^17^, with the mode set to “union.” For the *B. malayi* genome, RNAmmer v1.2^18^ identified 5 protein-coding genes (WBGene00228061, WBGene00268654, WBGene00268655, WBGene00268656, WBGene00268657) overlapping with predicted rRNAs. Reads overlapping with these genes were excluded from all gene-based analyses.

The sequencing reads for the *w*Bm comparisons were mapped to the *w*Bm assembly^19^ using Bowtie v0.12.9^20^. Counts for each *w*Bm gene were calculated by summing the sequencing depth per base pair, obtained using the DEPTH function of SAMtools v1.1^21^, for each of the unique genomic positions in each gene. This value was then divided by the average read length for the sample, yielding a read count value for each gene. Across all comparisons, read counts were normalized between each of the different samples through a conversion to transcripts per million (TPM).

The fold enrichment, *F*_*e*_, of reads conferred by the AgSS to its target organism compared to the other enrichment techniques was calculated as

$${F}_{e}=(\frac{{M}_{AgSS}}{{S}_{AgSS}})/(\frac{{M}_{poly(A)}}{{S}_{poly(A)}})$$

where the ratio of the number of reads mapped with AgSS capture (*M*_*AgSS*_) to the number of reads sequenced with AgSS capture (*S*_*AgSS*_) is divided by the ratio of the number of reads mapped with poly(A)-enrichment or the rRNA-, poly(A)-depletion (*M*_*poly* (*A*)_) to the number of reads sequenced with poly(A)-enrichment or the rRNA-, poly(A)-depletion (*S*_*poly* (*A*)_). By taking a ratio of the percentage of mapped reads for each enrichment technique, the fold enrichment value represents an amount of enrichment conferred by the AgSS when compared to alternative enrichment technique for a given sample. The fold enrichment values when comparing the efficacy of AgSS capture on poly(A)-enriched libraries to total RNA libraries were calculated similarly as

$${F}_{e}=(\frac{{M}_{AgSS-poly(A)}}{{S}_{AgSS-poly(A)}})/(\frac{{M}_{AgSS-totalRNA}}{{S}_{AgSS-totalRNA}})$$

where ${M}_{AgSS-poly(A)}$ and ${S}_{AgSS-poly(A)}$ represent the mapped and sequenced reads from poly(A)-enriched AgSS capture libraries, while ${M}_{AgSS-totalRNA}$ and ${S}_{AgSS-totalRNA}$ represent the mapped and sequencing reads from AgSS capture from the total RNA libraries.

## Results

### Agilent SureSelect probe G + C content and specificity

The G + C content of the *B. malayi* AgSS probes, our largest set of designed probes, was assessed to determine whether the G + C content of a probe impacted its ability to capture its target gene. For any given gene in the *B. malayi* genome, the G + C content of its set of probes ranged from 19.2–66.2%, with an average G + C content of 38.6% (Supplementary Fig. 1). For each G + C content, in increments of 1%, the average ratio of the AgSS TPM to the poly(A)-selected TPM average ratio of all genes with probe sets of that specified G + C content were calculated and compared. Between 26–55%, there were >10 genes with probe sets at each of the G + C increments. At these percentages and across six different samples, the relationship between the AgSS TPM to poly(A)-selected TPM remained linear, indicating that the ability of a probe set to capture its gene set is not largely affected by G + C content (Supplementary Fig. 2).

Across the six *B. malayi* samples, <2,004 probes were invalid, which accounts for ~1.2% of the 167,997 designed probes for *B. malayi*. For each of the samples, an invalid probe was defined as a probe whose intended capture positions had a larger read count in the poly(A)-selected sample relative to its AgSS counterpart.

### Comparison of poly(A)-enriched AgSS to only poly(A)-enriched libraries for *B. malayi*

**Figure 1:**
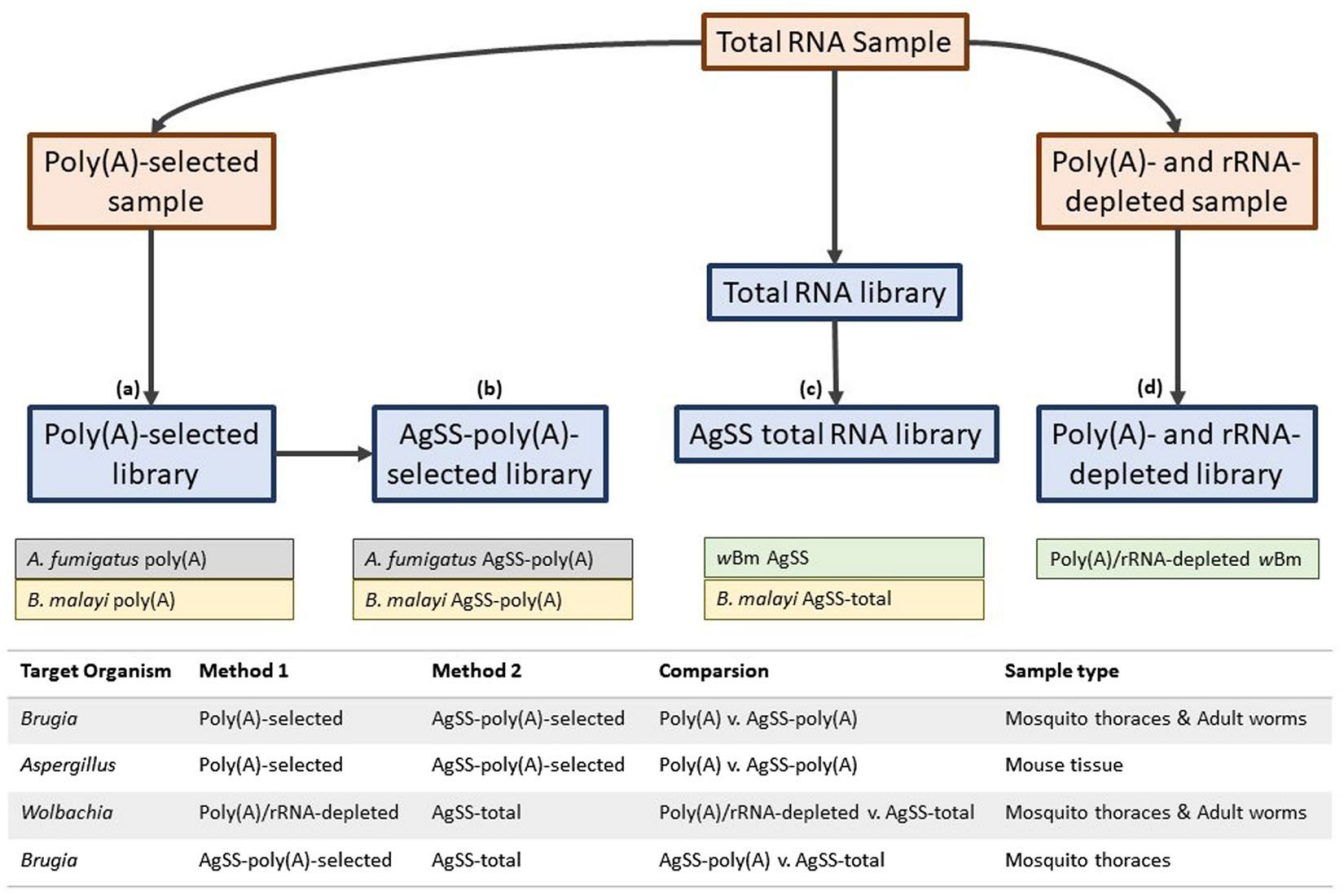
This schematic illustrates the sample (auburn rectangle) and library (blue rectangle) preparation workflow to generate the libraries that were loaded on the Illumina sequencer. (**a**) For *B. malayi* and *A. fumigatus*, a poly(A)-selected sample was created from an aliquot of total RNA that was used to create a poly(A)-selected library. (**b**) The *B. malayi* or *A. fumigatus* AgSS baits were subsequently used to capture the targeted RNA from poly(A)-selected libraries. (**c**) For AgSS-enriched *w*Bm libraries, an RNA library was constructed from an aliquot of total RNA that underwent targeted enrichment with the *Wolbachia* AgSS baits. Unlike the eukaryotic enrichments, the bacterial AgSS capture is performed on total RNA. For a limited number of libraries described in the text, an RNA library was constructed from an aliquot of total RNA (i.e. without poly(A)-enrichment) that underwent targeted enrichment with the *Brugia* AgSS baits. (**d**) For poly(A)/rRNA-depleted libraries enriched for *w*Bm, an aliquot of total RNA from either mosquito thoraces or adult nematodes was enriched for bacterial mRNA by removing Gram-negative and human rRNAs with two RiboZero removal kits and polyadenylated RNAs with DynaBeads.

Poly(A)-enriched, AgSS libraries were compared to only poly(A)-enriched libraries from six RNA samples originating from three different time points of the *B. malayi* vector life stages (18 hpi, 4 dpi, and 8 dpi) (Fig. 1a). Across these six samples, the poly(A)-enrichment alone yielded 0.38–23.35% of reads mapping to the *B. malayi* genome, with 61.25–82.51% of reads mapping to the *A. aegypti* genome. Of the poly(A)-enriched libraries captured using the AgSS baits, 56.14–81.52% of reads mapped to the *B. malayi* genome, with 2.28–24.02% of reads mapping to the *A. aegypti* genome (Supplementary Dataset 1, Supplementary Fig. 3). The fold enrichment conferred to libraries captured with AgSS was found to be inversely proportional to the number of reads mapped to its poly(A)-enrichment only counterpart, such that experiments with the fewest reads mapped in the poly(A)-enrichment had the highest fold enrichment with the AgSS capture. The 18 hpi samples, which have the lowest percentage of mapped reads to the *B. malayi* genome has the greatest fold enrichment (110–146x) while the 8 dpi samples, with the highest percentage of *B. malayi* mapped reads, has the lowest fold enrichment (3–4x).

**Figure 2:**
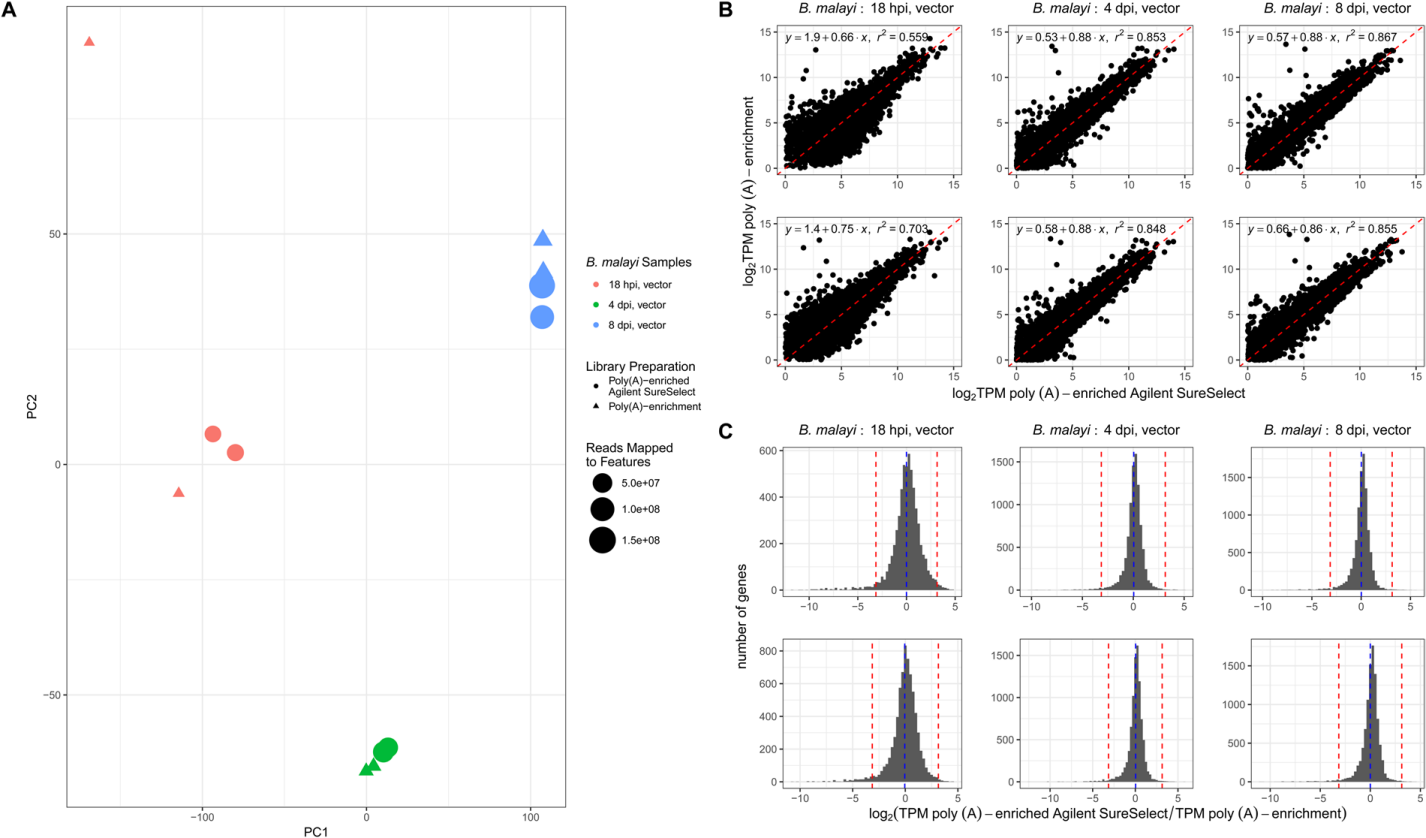
Poly(A)-enriched transcriptomes were compared to the poly(A)-enriched AgSS *B. malayi* transcriptomes for the 18 hpi, 4 dpi, and 8 dpi vector life stages. (**A**) A principal component analysis (PCA) plot was generated using the log_2_ TPM values for the poly(A)-enriched only (triangles) and poly(A)-enriched AgSS (circles) *B. malayi* from 18 hpi (red), 4 dpi (green), and 8 dpi (blue) vector samples. The size of each symbol denotes the number of reads mapped to protein-coding genes for each sample. The samples cluster based on *B. malayi* sample rather than by the enrichment method, indicating the poly(A)-enriched AgSS does not substantially differ from its poly(A)-enrichment only counterpart in representing the *B. malayi* transcriptome for the shown samples. Samples with the lowest number of reads (e.g. the 18 hpi samples) cluster further from their replicates than expected, likely attributed to an insufficient number of reads and indicating that enrichment is most desirable in these situations. (**B**) For each sample, the log_2_ TPM values for genes in the poly(A)-enriched AgSS samples were plotted against the log_2_ TPM values for genes in the poly(A)-enrichment only samples when expression was observed with both enrichment methods for the sample. Genes with similar expression in the AgSS and the poly(A)-enrichment samples are expected to lie close to the identity line (x = y; red). Genes whose expression values are more elevated in the poly(A)-enriched AgSS sample compared to the poly(A)-enrichment sample lie below the identity line while genes more elevated in the poly(A)-enrichment compared to the poly(A)-enriched AgSS lie above the identity line. (**C**) For all genes expressed using both enrichment methods for each sample, the frequency distribution of the log_2_-transformed ratio of the poly(A)-enriched AgSS TPM to the poly(A) enrichment TPM was plotted. Genes with log_2_ ratio values > 0 have higher expression values in the poly(A)-enriched AgSS sample while log_2_ ratio values < 0 are representative of genes with higher expression values with the poly(A) enrichment. Significantly biased genes were defined as those differing by >3 standard deviations (red) from the mean of the log_2_-transformed ratio values (red). Across each comparison, the number of genes with significantly elevated expression in the AgSS comprised at most 0.31% of the total number of *B. malayi* protein-coding genes while the number of genes with significantly elevated expression in the poly(A)-enrichment samples comprised at most 1.52% of *B. malayi* protein-coding genes.

A principal component analysis (PCA) using the log_2_-TPM values for the 11,085 predicted *B. malayi* protein-coding genes reveals that the *B. malayi* samples cluster based on life stage rather than library preparation (Fig. 2A), suggesting that the capture does not introduce a systematic bias to the transcriptome of a sample. The exceptions are the 18 hpi vector samples, which we attribute to the poly(A)-enrichment only samples having an insufficient number of reads mapped to features (143,078 and 212,342 reads, accounting for <20 reads/gene assuming an equal distribution) to accurately represent the transcriptome. Supporting this, in the 18 hpi samples, an average of 2,460 protein coding genes (22.18% of the *B. malayi* protein coding genes) had reads mapping to them in the poly(A)-enriched AgSS samples but not its poly(A)-enriched counterpart, while in the 4 dpi and 8 dpi samples, an average of 952 (8.58%) and 566 (5.09%) genes, respectively, had reads mapped in the poly(A)-enriched AgSS libraries but not the poly(A)-enriched libraries. Collectively, across all 6 samples, 392 unique genes were detected in only the poly(A)-enrichment, but not its poly(A)-enriched AgSS counterpart in at least one comparison. The AgSS probe design was based on an older version of the annotation compared to the version used for feature calling, and as such, 91 of these 392 genes were not covered by a probe.

The log_2_ TPM values from the poly(A)-enriched AgSS and the poly(A)-enriched libraries had a positive linear correlation with r^2^ values ranging from 0.559–0.867 (Fig. 2B), with the 4 dpi and 8 dpi correlations being the strongest (Fig. 2B), for the reasons discussed above, namely that fewer reads were recovered from the 18 hpi vector samples without AgSS enrichment. To identify individual genes in which the library preparations significantly affected the calculated TPM, the log_2_ ratio of the poly(A)-enriched AgSS TPM relative to the poly(A) enrichment TPM for each gene was calculated (Fig. 2C). Genes with relatively similar expression levels in the two library preparations would have a log_2_ ratio of 0 while genes more highly expressed in the poly(A)-enriched AgSS preparation would be >0 and genes more highly expressed in the poly(A) enrichment would be <0. Genes with a log_2_-ratio value of >3 standard deviations from the mean were defined as having significantly altered levels of expression between the two libraries. Across all six comparisons, the number of genes with significantly higher TPM values in the AgSS libraries never accounted for >0.31% (<34 genes) of all protein-coding genes in the *B. malayi* genome. The number of genes with significantly higher TPM values in the poly(A)-enrichment ranged from 0.93–1.52% (103–168 genes) of all protein-coding genes in *B. malayi* across the six comparisons **(**Supplementary Dataset 1, Supplementary Fig. 3).

### Comparison of poly(A)-enriched AgSS to only poly(A)-enrichment libraries for *A. fumigatus*

The *A. fumigatus* AF293 transcriptome from poly(A)-enriched AgSS and poly(A)-enriched only libraries were compared for 11 RNA samples from immunocompromised mice or neutropenic mice infected with *A. fumigatus* AF293 (Fig. 1a). Of the 11 samples, 6 samples originated from 3 replicates of immunocompromised mice taken 2 dpi and 4 dpi with *A. fumigatus*. The remaining 5 samples consist of two replicates of neutropenic mice taken 4 dpi with *A. fumigatus* and three replicates of neutropenic mice taken 7 dpi with *A. fumigatus*.

Across all 11 samples, the total percentage of reads mapping to *A. fumigatus* from the poly(A)-enriched samples was consistently ≤0.02%, while 0.30%–11.74% of reads mapped in the poly(A)-enriched AgSS samples. In the poly(A)-enrichment only samples, a maximum of 27,531 reads mapped to *A. fumigatus* AF293, accounting for <3 reads/gene assuming an even distribution. As such, comparisons between AgSS and poly(A)-enriched libraries are not possible.

There are 4,168–7,387 genes (41.68–76.70%) that are identified only in the poly(A)-enriched AgSS libraries but not the poly(A)-enriched libraries, compared to the maximum of 52 genes identified only in the poly(A)-enriched libraries across all *A. fumigatus* comparisons **(**Supplementary Dataset 2, Supplementary Fig. 4**)**. For all 11 comparisons, using the AgSS methodology conferred a 614x to 1,212x fold enrichment of *A. fumigatus* AF293 reads relative to the poly(A)-enrichment, going from data that was unamenable to a traditional transcriptomics analysis to obtaining data where robust statistical tests can be performed. The proportion of reads mapping to features relative to the total number of reads mapped is consistent between samples of the same library preparation, with *A. fumigatus* poly(A)-enriched AgSS samples ranging from 52.23–54.87% and the poly(A)-enrichment samples ranging from 45.89–51.22%.

### Comparison of total RNA AgSS to rRNA-, poly(A)-depleted libraries for the bacterial *Wolbachia* endosymbiont *w*Bm

With both of the previously discussed biological systems, we tested the effectiveness of the AgSS platform to study eukaryote-eukaryote host-pathogen interactions. By enriching for *w*Bm reads in *B. malayi* samples, we sought to test the effectiveness of AgSS libraries in extracting prokaryotic reads. The standard protocol for AgSS capture in eukaryotes relies on capturing targets from poly(A)-enriched libraries, but for prokaryotic samples the capture is performed on total RNA libraries. These total RNA AgSS libraries were compared to rRNA-, poly(A)-depleted library preparations from nine *B. malayi* samples taken at various stages of the life cycle to analyze the transcriptome of its *Wolbachia* endosymbiont, *w*Bm (Fig. 1b). Three RNA samples were obtained from the mammalian portion of the *Brugia* lifecycle where worms are large enough to be physically separated from the mammalian tissue. These RNA samples generate predominantly *Brugia* reads with a minority of *Wolbachia* reads. Of these three samples, one sample was recovered at 24 dpi, representative of immature female worms while the other two samples are from two reproductively mature adult females recovered 3 months post-infection. The other six samples are from the vector portion of the *B. malayi* lifecycle where tripartite RNA samples are obtained, consisting of predominantly mosquito reads, with a smaller percentage of *Brugia* reads, and an even smaller percentage of *Wolbachia* reads. These six samples consist of two *A. aegypti* replicates each taken at18 hpi, 4 dpi, and 8 dpi with *B. malayi*, representative of the L1 through early L3 *B. malayi* life stages. Of the six samples taken from the vector life stages, at most 1,552 reads (<0.01% of sequenced reads) mapped to the *w*Bm genome from the rRNA-, poly(A)-depleted samples, of which <730 reads were identified as mapping to protein-coding genes for an average of <1 read mapped/gene (Supplementary Dataset 3, Supplementary Fig. 5). In contrast, the rRNA-, poly(A) depleted adult female *B. malayi* samples taken from the mammalian host had a minimum of 17,107 reads mapping to *w*Bm protein-coding genes, equating to ~20 reads/gene while the 24 dpi sample had 246,549 reads mapped to protein-coding genes, equating to ~294 reads/gene.

The AgSS-enriched samples had an increased number of *w*Bm-mapping reads for all 9 samples, with 20.4–37.4 million reads (18.68–32.53%) mapping to *w*Bm genes for the three gerbil samples and 0.2–4.0 million reads (0.46–6.43% of sequenced reads) mapping to *w*Bm genes for the mosquito vector samples. The AgSS libraries from gerbil samples were 20–41x fold enriched for *w*Bm reads relative to the rRNA-, poly(A)-depleted libraries while the libraries from vector samples were 353–2,242x fold enriched. The proportion of *w*Bm reads mapped to protein-coding genes were consistently higher in the AgSS-capture sample for both the gerbil and vector samples, ranging from 88.31–92.44% and 92.04–92.81%, respectively, compared to the 10.62–20.11% and 45.21–58.73% observed in the rRNA-, poly(A)-depleted samples. This difference in the number of *w*Bm reads mapping to protein-coding genes can be partially attributed to a greater number of reads mapping to rRNAs in the rRNA-, poly(A)-depleted samples, with 18.73–33.82% of *w*Bm mapped reads mapping to rRNAs, compared to 0.1–2.1% observed in the total RNA, AgSS samples (Supplementary Dataset 3, Supplementary Fig. 5).

**Figure 3:**
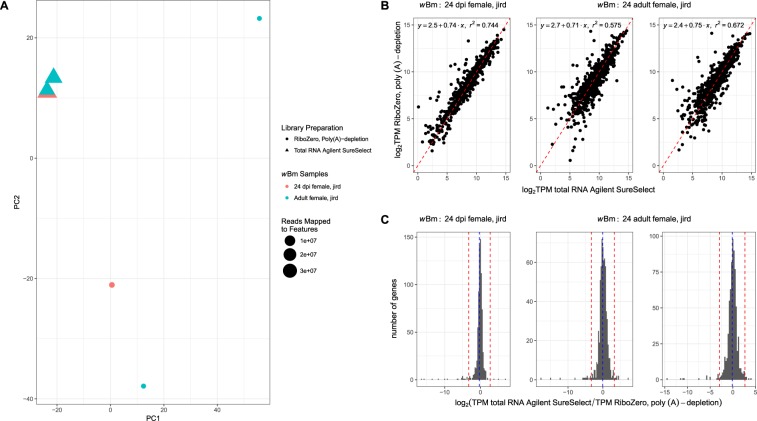
The rRNA-, poly(A)-depleted transcriptomes were compared to the total RNA AgSS *w*Bm transcriptomes for the female 24 dpi and adult *B. malayi* life stages. (**A**) A principal component analysis of the rRNA-, poly(A)-depleted (circle) and total RNA AgSS (triangle) *w*Bm transcriptome from three samples of the *B. malayi* gerbil life stage was conducted, with one sample from female 24 dpi *B. malayi* (red) and two samples from adult female *B. malayi* (blue). The size of each point is relative to the number of reads mapped to protein-coding genes for each sample. In this instance, the samples no longer cluster based on the sample type. Instead, the samples distinctly cluster apart based on enrichment method, most likely due to the smaller number of reads present in samples lacking the AgSS capture. Consistent with this, the sample with the fewest reads, one of the rRNA-, poly(A)-depleted adult females, is most distant. The three samples enriched using the AgSS cluster together, possibly indicating the similarity of *w*Bm transcriptomes originating from female 24 dpi and adult *B. malayi*. (**B**) For each sample, the log_2_ TPM values for genes detected with the AgSS capture on total RNA were plotted against the log_2_ TPM values for genes detected with the rRNA-, poly(A)-depletion when a gene was detected in both enrichments. Genes with similar expression in both the total RNA AgSS and the rRNA-, poly(A)-depleted samples are expected to lie close to the identity line (x = y; red). Genes whose expression values are more elevated in the total RNA AgSS compared to the rRNA-, poly(A)-depletion lie below the identity line while genes more elevated in the rRNA-, poly(A)-depletion compared to the total RNA AgSS lie above the identity line. (**C**) The frequency distribution log_2_-transformed ratio of the total RNA AgSS TPM to the rRNA- and poly(A)-depleted TPM for each gene detected in both enrichment methods was plotted. The blue, dashed line indicates the mean log_2_-transformed ratio while the red dashed line marks three standard deviations from the mean log_2_-transformed ratio. Genes with log_2_ ratio values >3 standard deviations above or below the mean were marked as biased towards the total RNA AgSS or the rRNA-, poly(A)-depletion, respectively. Across all 9 comparisons, an average of ~0.4 genes were detected with significantly higher expression with the total RNA AgSS, while an average of ~4.0 genes had significantly higher expression in the rRNA-, poly(A)-depleted samples.

A principal component analysis using the TPM values of the three gerbil life stage comparisons demonstrates that individual samples segregate based on library preparation (Fig. 3A). All three of the total RNA AgSS samples are clustered close together, while the rRNA-, poly(A)-depleted samples are further distributed, likely due to the insufficient coverage of the *w*Bm transcriptome in the absence of AgSS enrichment. The mosquito vector life stage samples were not used for the principal component analysis due to an insufficient number of reads mapping in the poly(A)-depletion portion of the comparison to compare the two enrichment methods. Like the *B. malayi* comparisons, the log_2_ TPM values from the *w*Bm AgSS libraries and the rRNA- and poly(A)-depletion libraries made from mammalian life stages had a positive linear correlation with r^2^ values ranging from 0.575–0.744 (Fig. 3B).

A total of 45 unique genes (~5.4% of protein-coding genes in *w*Bm) were detected in at least one of the three gerbil comparisons in the sample treated using the rRNA-, poly(A)-depletion libraries but not its total RNA AgSS counterpart. Of these 45 genes, 41 were not covered by any of the AgSS probes while the remaining 4 genes had <11% of their length covered by a probe. To identify genes with significantly greater expression in one enrichment method over the other, a cutoff of three standard deviations from the log_2_ ratio of the AgSS TPM to the rRNA-, poly(A)-depleted TPM was used (Fig. 3C). Genes with a log_2_ ratio of >3 standard deviations below the average log_2_ ratio were determined to have significantly higher expression in the rRNA-, poly(A)-depletion sample while genes with a log_2_ ratio >3 standard deviations above the average, were identified as having significantly higher expression in the total RNA AgSS sample. Across the three comparisons, a total of 20 unique genes had TPM values significantly higher in the rRNA-, poly(A)-depleted sample compared to the AgSS sample in at least one comparison. Of these genes, 14 genes were not covered by any probe, four genes had <17% of their length covered by a probe, and the remaining two genes are covered in the entirety of their length by the probe design. Additionally, there were 152 unique genes (~18.1% of protein-coding genes in *w*Bm) detected in the total RNA, AgSS but not the poly(A)-depletion in at least one of the comparisons. Of the 152 genes, 114 had average TPM values in the bottom quartile of the expressed genes across the three AgSS samples, highlighting the ability of the AgSS preparation to detect low abundance transcripts.

### Comparison of AgSS *B. malayi* transcriptome data from total RNA and poly(A)-enriched samples

For eukaryote transcriptome experiments, the AgSS platform is typically preceded by construction of a poly(A)-selected library that is then hybridized to the bait probes. The poly(A)-enrichment step serves to remove high abundance, non-mRNA transcripts, such as rRNAs, tRNAs, and other ncRNAs. For bacterial samples, libraries must be constructed on total RNA since bacterial mRNAs lack the poly(A) tails present in eukaryotic transcripts. For eukaryote-prokaryote transcriptomic experiments, it may be advantageous to use the AgSS platform for both bacteria and eukaryotes on the same single library constructed from total RNA, especially for clinical samples where RNA may be limiting. Therefore, we sought to compare the AgSS enrichment on a library constructed from total *B. malayi* RNA with one constructed following poly(A)-enrichment using two samples taken from the mosquito vector portion of the *B. malayi* life stages at 18 hpi and 8 dpi (Fig. 1c).

**Figure 4:**
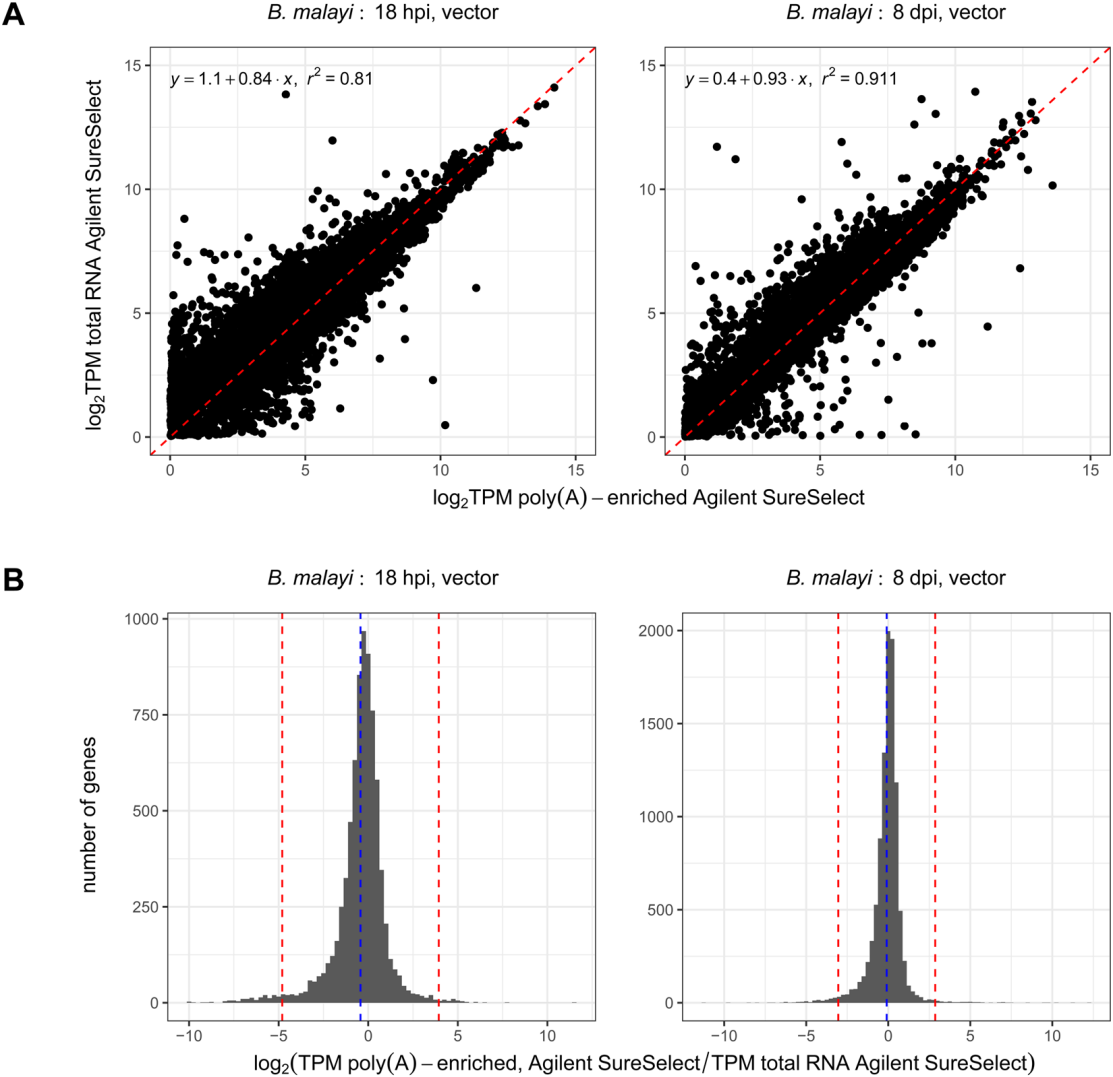
The poly(A)-enriched AgSS transcriptome was compared to the total RNA AgSS *B. malayi* transcriptomes for the 18 hpi and 8 dpi vector life stages. (**A**) For both the 18 hpi and 8 dpi vector samples, the log_2_ TPM values for each *B. malayi* gene detected in both the poly(A)-enriched AgSS and the total RNA AgSS were plotted against one another. Most genes in both plots fall close to the identity line (y = x; red), indicating their similar expression values in both the poly(A)-enriched AgSS and the total RNA AgSS libraries. (**B**) A histogram of the log_2_ ratios of the poly(A)-enriched AgSS TPM to the total RNA AgSS TPM was generated for each sample using all genes detected with both enrichment methods. The blue dashed line marks the average log_2_ ratio value while the red lines mark three standard deviations from the average log_2_ ratio. Genes with log_2_ ratio values >3 standard deviations above the average are marked as significantly elevated in the poly(A)-enriched AgSS while genes with log_2_ ratio values >3 standard deviations below the average are marked as significantly elevated in the total RNA AgSS samples. For both the library preparations, ≤1.4% (155 genes) of protein-coding genes are biased towards a single enrichment method, indicating no significant bias towards an enrichment.

For both the 18 hpi and 8 dpi time points, most genes yield comparable log_2_ TPM values between the two enrichment methods (Fig. 4A) with <1.5% of genes having a bias towards a particular library construction protocol (Fig. 4B). However, despite the 8 dpi comparison yielding a similar number of genes detected unique to each enrichment method, in the 18 hpi sample comparison, 1,033 genes (9.3% of all protein-coding genes) were detected only in the poly(A)-enriched AgSS library while 323 (2.91%) were only detected with the total RNA AgSS library **(**Supplementary Dataset 4, Supplementary Fig. 6). In both cases, a majority of the genes detected in only one library construction method were within the bottom 20% of expressed genes in the sequencing data collected, with 815 genes (78.9%) in the selection conducted with the total RNA library and 233 genes (72.14%) in the selection conducted with the poly(A)-enriched library. Similarly, in the 8 dpi sample comparison, 2.35% (261 genes) of *B. malayi* protein-coding genes were identified only in the AgSS poly(A)-enriched library while 1.71% (189 genes) were identified only in the target AgSS total RNA library. Again, in both cases, a majority of the genes detected in only one library construction method were within the bottom 20% of expressed genes in the sequencing data collected, with 236 genes (90.42%) in the selection conducted with the poly(A)-enriched RNA library and 179 genes (94.71%) in the selection conducted with the total RNA library. Based off these results, both AgSS methods can detect low abundance transcripts, but the poly(A)-enriched AgSS was able to select for a greater number of these transcripts in both comparisons.

The percentage of reads mapping to the *B. malayi* genome were greater in the poly(A)-enriched AgSS samples compared to the total RNA AgSS samples, with 56.14% and 80.13% of reads mapping to the *B. malayi* genome, compared to 22.96% and 69.84% of reads mapped for the 18 hpi and 8 dpi samples respectively. The poly(A)-enriched AgSS samples were enriched for reads mapping to *B. malayi* by 2.4x for the 18 hpi sample and 1.1x for the 8 dpi sample relative to the total RNA AgSS. Additionally, in the 18 hpi and 8 dpi poly(A)-enriched AgSS samples, 46.63% and 46.35% of their mapped reads mapped to protein-coding genes while in the total RNA AgSS samples only 20.22% and 32.21% mapped to protein-coding genes (Supplementary Dataset 4, Supplementary Fig. 6). This is explained by the increased number of multi-mapping reads observed in the total RNA AgSS samples, with 70.29% and 39.29% of the total number of mapped reads in the 18 hpi and 8 dpi vector samples multi-mapping to the *B. malayi* genome. In comparison, only 5.93% and 5.42% of mapped reads were multi-mapping in the 18 hpi and 8 dpi poly(A)-enriched AgSS samples. A total of 80.82% and 69.86% of these multi-mapping reads in the total RNA AgSS 18 hpi and 8 dpi vector samples were found to map to the *B. malayi* rRNAs compared to only 13.76% and 1.56% in the poly(A)-enriched AgSS samples (Supplementary Dataset 4, Supplementary Fig. 6). The AgSS platform on poly(A)-enriched RNA was able to obtain both a higher percentage of *B. malayi* mapped reads and a higher percentage of *B. malayi* reads mapping to protein-coding genes. Therefore, we recommend the use of the AgSS enrichment on poly(A)-enriched libraries for eukaryotic systems whenever possible.

### Recovering the transcriptome of the major organism from libraries prepared with the AgSS of the minor organism

We explored whether sequencing data from both the major and minor organism could be accurately obtained using only data collected from a AgSS capture of the minor organism for eukaryote-eukaryote dual-transcriptomics experiments. Using the sample with the highest fold enrichment conferred by the AgSS platform, an immunocompromised mouse at 2 dpi, the host mouse transcriptome was compared between two libraries enriched using only the poly(A)-enrichment versus a poly(A)-enriched library that was AgSS-captured for *A. fumigatus* transcripts. When plotting the TPM values of two library preparations against one another (Supplementary Fig. 7**)**, the TPM values share a positive linear correlation with a r^2^ of 0.85, similar to the correlation values observed in the *B. malayi* comparisons (Fig. 2B), indicating that the use of the *A. fumigatus* AgSS did not appear to largely impact the mouse transcriptome. However, of the 39,179 mouse genes, 5,424 (13.8%) were detected in only the polyA-library but not the corresponding AgSS-captured library. In comparison, only 128 mouse genes were detected only in the *A. fumigatus* AgSS library but not the poly(A)-library. While the transcripts captured seem to have the same expression profile, >10% of the genes were missed, and thus, we would recommend only assessing the organism for which the AgSS capture system was designed if the goal is to obtain the complete transcriptional profile of the major member.

## Discussion

With multi-species RNA-Seq experiments emerging at the forefront of experimental designs for transcriptome analyses, novel methods are being developed to more efficiently extract the transcriptomes for multiple organisms from a single sample. By comparing the transcriptomes generated with standard enrichment methods (e.g. poly(A)-enrichment or rRNA-/poly(A)-depletion) to the AgSS platform, we sought to independently validate the use of the AgSS platform in enriching for transcripts originating from a specific organism of interest while minimizing any type of library bias on the transcriptome. When analyzing the *B. malayi* transcriptomes of samples prepared with or without the AgSS enrichment, most genes could be detected using both library preparations. However, the samples prepared using the AgSS system enriched the number of reads mapped by 3x–146x compared to samples enriched only with a poly(A)-enrichment enabling the consistent detection of a greater number of genes, most of which were low abundance transcripts. In the *A. fumigatus* comparisons, the poly(A)-enrichment by itself was unable to extract a sufficient number of *A. fumigatus* reads to accurately represent the transcriptome of the sample. When the poly(A)-enrichment was supplemented with AgSS capture, the enrichment of *A. fumigatus* reads was increased 614–1,212x. When using the AgSS instead of the rRNA-, poly(A)-depletion preparation for the *Wolbachia* endosymbiont *w*Bm transcriptome, the AgSS samples were enriched for *w*Bm mapped reads by 20–41x in the gerbil life cycle samples and 353–2,242x in the vector life cycle samples.

**Figure 5:**
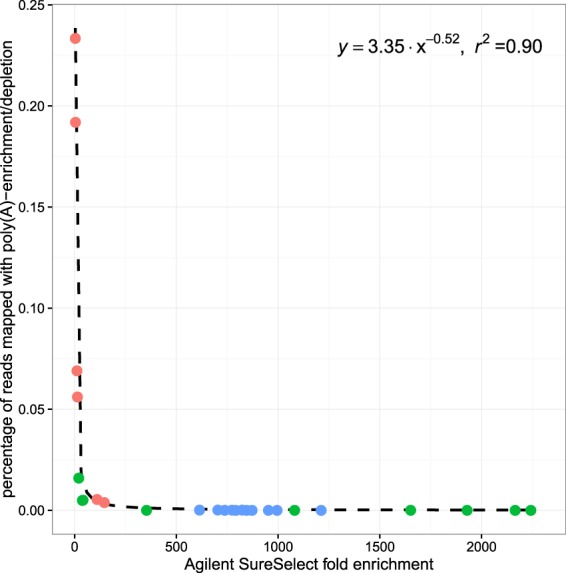
The fold enrichment value for each sample used in the poly(A)-enrichment/depletion versus AgSS comparisons for *B. malayi* (red), *A. fumigatus* (blue), and *w*Bm (green) was calculated by taking the ratio of the percentage of AgSS reads mapped to the percentage of poly(A)-enrichment/depletion reads mapped. The relationship between these fold enrichment values and the percentage of the poly(A)-enrichment/depletion reads mapped to the reference genome can be fitted to a power law. The fold enrichment conferred using the AgSS platform exponentially increases with the exponential decrease of the percentage of reads mapped using the standard enrichment/depletion methods up to an ~1300-fold enrichment.

For each of these samples, if the fold enrichment for the number of mapped reads in the poly(A)-enriched or total RNA AgSS is plotted against the percentage of reads mapped in its poly(A)-enrichment or poly(A), rRNA depletion counterpart, the data follows a power law (Fig. 5). As expected, the fold enrichment obtained with the AgSS capture is inversely proportional to the percentage of mapped reads obtained with the currently used enrichment methods of poly(A)-enrichment to capture eukaryotic RNA, or rRNA- and poly(A)-depletion to capture bacterial RNA from multi-species samples, indicating samples with a lower relative abundance of RNA from the target organism yield the greatest fold enrichment.

While the AgSS can enrich a sample for transcripts of interest, the platform requires probes designed for each gene to avoid any enrichment bias. Most instances of the AgSS failing to detect a gene that was detected in samples enriched using only the poly(A)-enrichment or rRNA-, poly(A)-depletion was due to a lack of designed probes for the gene. Because the *B. malayi* annotation is currently being updated, the baits were designed with an older annotation than the one used for feature counting. Similarly, the bait design for *w*Bm was based on the annotation published by Foster *et al*.^19^ while the annotation used for feature counting originated from GenBank (NC_006833.1). In both cases, most genes present only in the annotation used for the bait design were not detected with the AgSS platform.

These results illustrate the power and advantages of using probe-based enrichment to facilitate the analysis of samples that are the most insightful, like those in animal tissue at a biologically relevant multiplicity of infection. The AgSS platform serves as a method to extract reads from a secondary or tertiary organism in a sample as well as to detect low abundance transcripts. Using this platform, we successfully extracted a sufficient number of reads to represent the *B. malayi* and *w*Bm transcriptomes in the vector portion of the *B. malayi* life cycle, the *A. fumigatus* transcriptome in a mouse infection, and the *w*Bm transcriptome in the mammalian portion of the *B. malayi* life cycle. Provided a transcriptome experiment has an adequate bait design, we believe the AgSS platform to be ideal and necessary in enriching samples for multi-species transcriptomic experiments, especially those in which secondary organisms are of low abundance.

## Electronic supplementary material

Supplementary Information

Supplementary Dataset 1

Supplementary Dataset 2

Supplementary Dataset 3

Supplementary Dataset 4

## Data Availability

The data set(s) supporting the results of this article are available in the Sequence Read Archive (SRA) repository. The *B. malayi* datasets are available in SRP068692, the *w*Bm datasets are available in SRP068711, and the *A. fumigatus* datasets are available in PRJNA421149.
