# Supplementary material for: Targeted enrichment outperforms other enrichment techniques and enables more multi-species RNA-Seq analyses
Source: Sci Rep. 2018 Sep 6;8:13377. doi: 10.1038/s41598-018-31420-7 (PMC6127098; doi:10.1038/s41598-018-31420-7)
Supplement: Supplementary file 1 — Supplementary Information [file 41598_2018_31420_MOESM1_ESM.docx]

# Targeted enrichment outperforms other enrichment techniques and enables more multi-species RNA-Seq analyses

Matthew Chung^1,2^, Laura Teigen^3^, Hong Liu^4^, Silvia Libro^5^, Amol Shetty^1^, Nikhil Kumar^1^, Xuechu Zhao^1^, Robin E. Bromley^1^, Luke J. Tallon^1^, Lisa Sadzewicz^1^, Claire M. Fraser^1,6^, David A. Rasko^1, 2^, Scott G. Filler^4,7^, Jeremy M. Foster^5^, Michelle L. Michalski^3^, Vincent M. Bruno ^1, 2^, Julie C. Dunning Hotopp^1,2,8^

# Supplementary Files


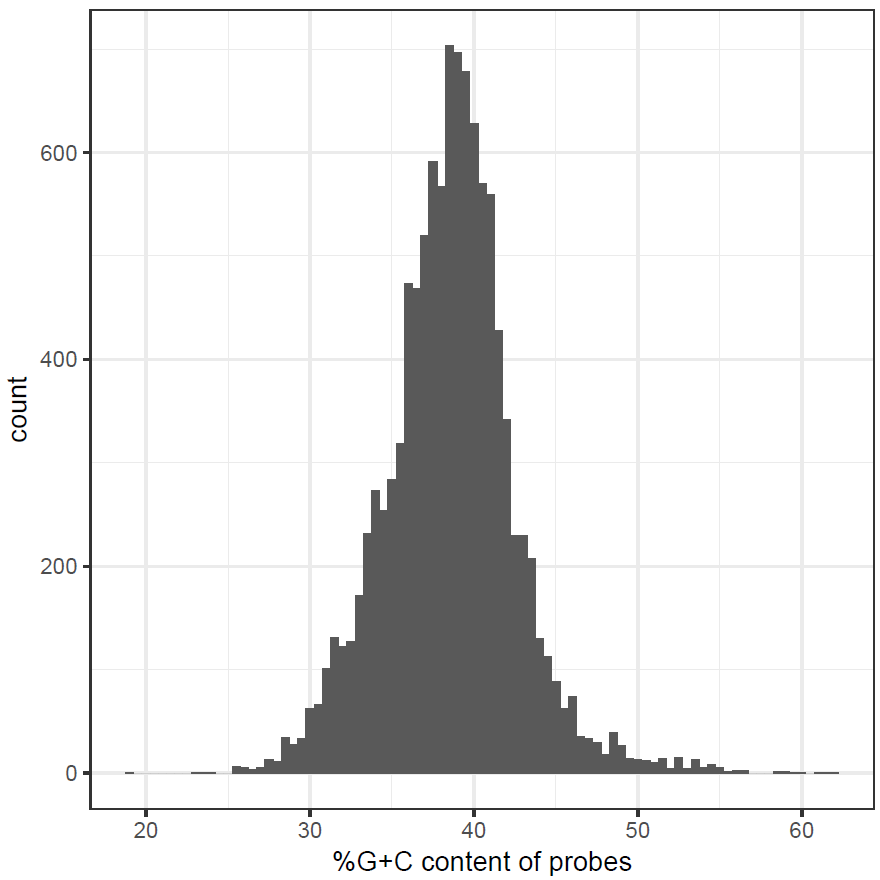


## Supplementary Figure 1. G+C content of Brugia malayi AgSS probes

For each gene in the *B. malayi* genome, the average G+C content for its set of probes was determined and plotted on a histogram. The average G+C content of the *B. malayi* probe sets is 38.6%, with a range of 19.2-66.2%.


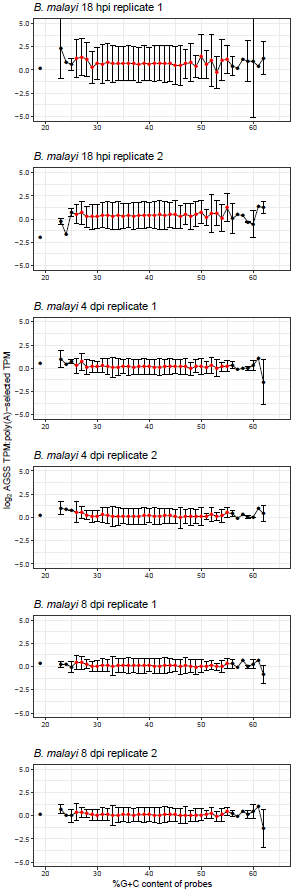


## Supplementary Figure 2. The impact of G+C content on capturing *Brugia malayi* genes

At increments of 1% G+C, probe sets for each gene were binned to a specific G+C content. Points colored in red, between 26-55% G+C content, indicate ≥10 genes have probe sets at that specific G+C content. Error bars indicate standard error of the mean for each G+C content. For any G+C content, the average ratio of the log_2_ AgSS TPM to the poly(A)-selected TPM for each protein-coding is calculated. Because 26-55% G+C content, the capture ability of a probe set is independent of its G+C content, as seen by the linear relationship between these points.


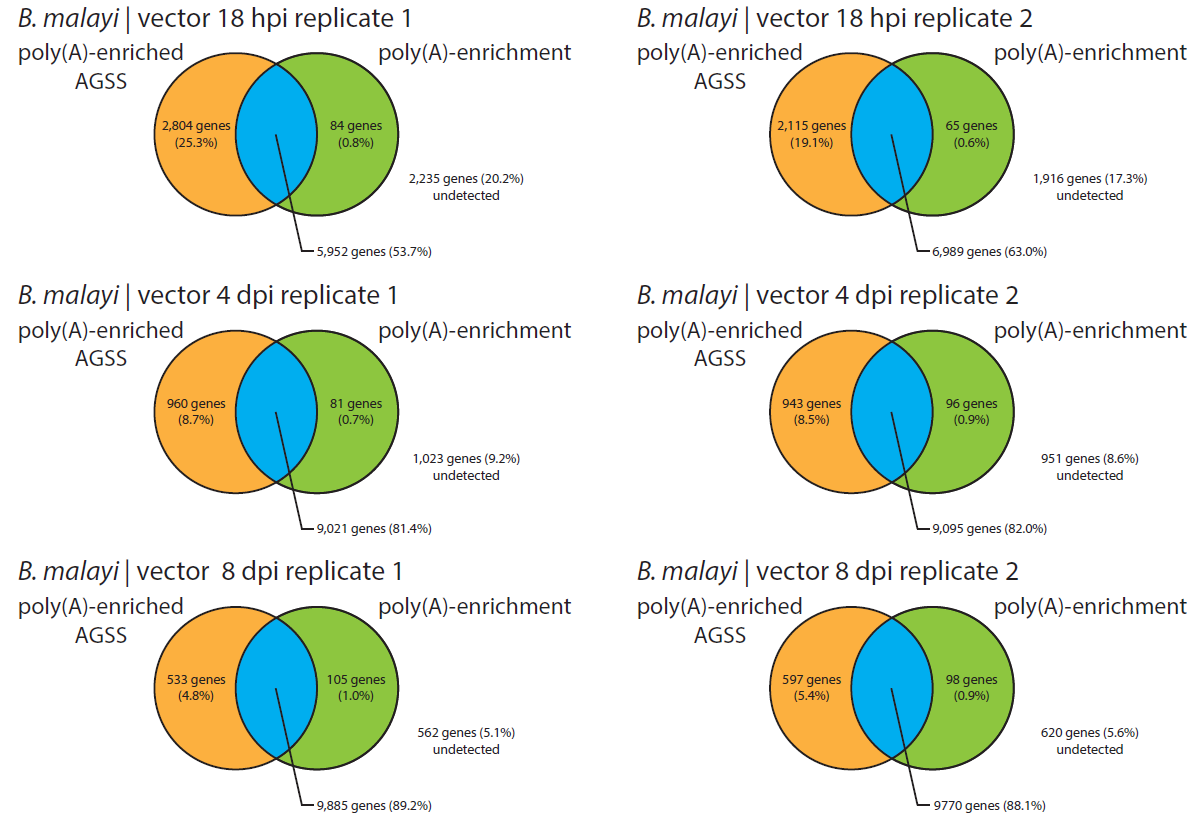


## Supplementary Figure 3. Genes identified only in either the poly(A)-enriched AgSS or poly(A)-enrichment libraries from *B. malayi* samples

For six *B. malayi* transcriptome comparisons, Venn diagrams were generated to display the number of genes able to be extracted using the poly(A)-enriched AgSS (orange), the poly(A)-enrichment (green), or both (blue). The numbers in parentheses indicate the proportion of total protein-coding genes identified in each gene subset.


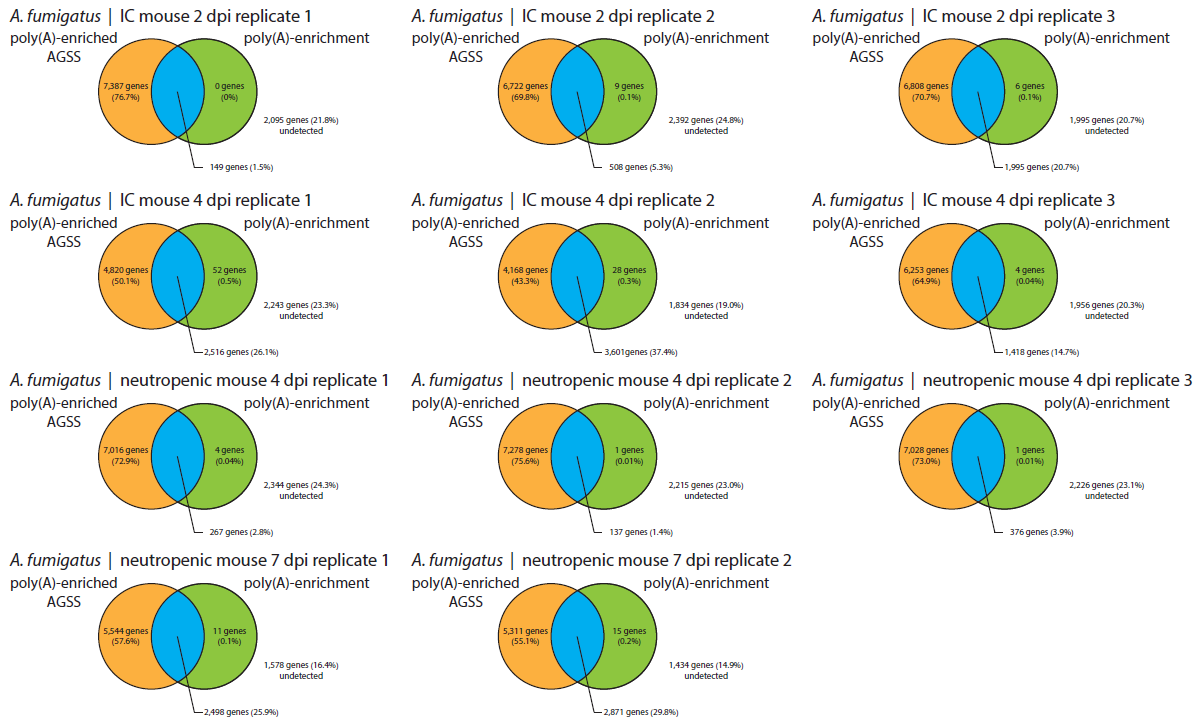


## Supplementary Figure 4. Genes identified only in either the poly(A)-enriched AgSS or poly(A)-enrichment libraries from *A. fumigatus* samples

For 11 *A. fumigatus* transcriptome comparisons, Venn diagrams were generated to display the number of genes able to be extracted using the poly(A)-enriched AgSS (orange), the poly(A)-enrichment (green), or both (blue). The numbers in parentheses indicate the proportion of total protein-coding genes identified in each gene subset.


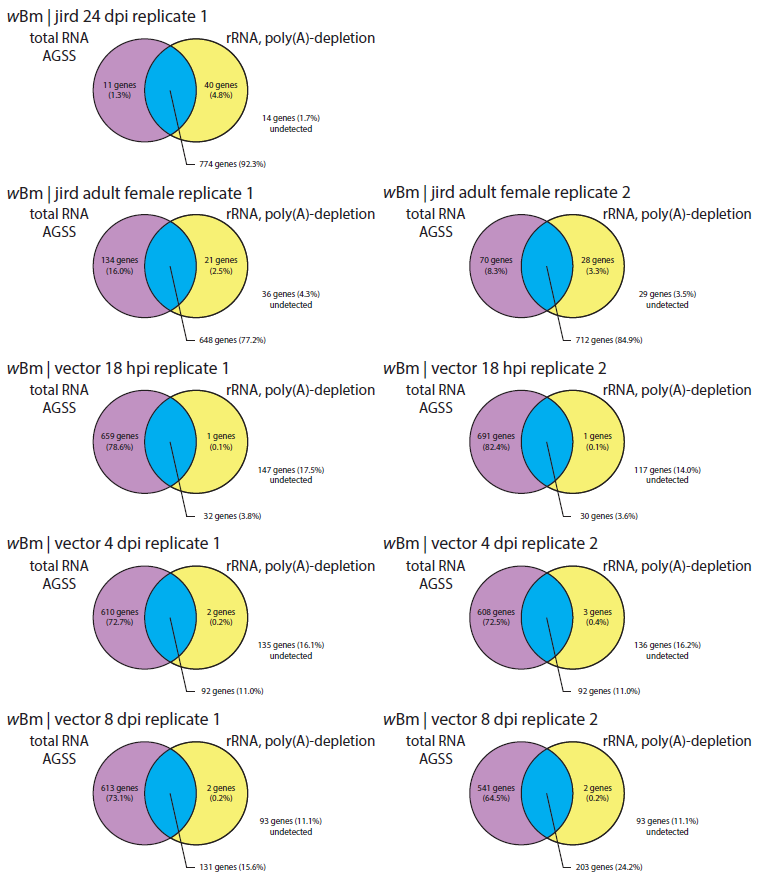


## Supplementary Figure 5. Genes identified only in either the total RNA AgSS or rRNA-, poly(A)-depletion libraries from *w*Bm samples

For nine *w*Bm transcriptome comparisons, Venn diagrams were generated to display the number of genes able to be extracted using the total RNA, AgSS (purple), the rRNA, poly(A)-depletion (yellow), or both (blue). The numbers in parentheses indicate the proportion of total protein-coding genes identified in each gene subset.


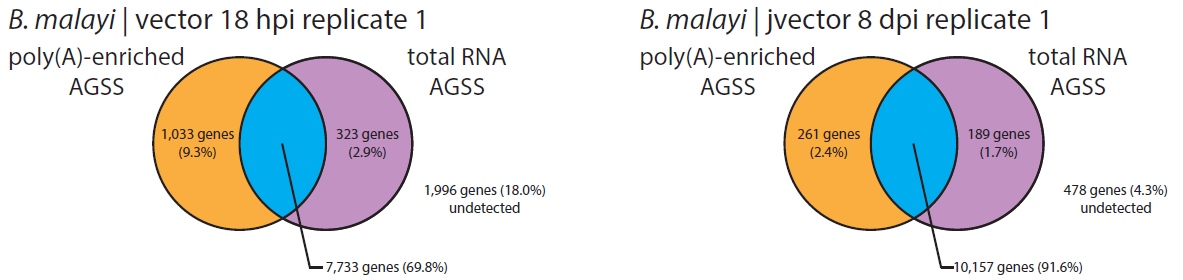


## Supplementary Figure 6. Genes identified only in either the total RNA AgSS or poly(A)-enriched AgSS libraries from *B. malayi* samples

For two *B. malayi* transcriptome comparisons, Venn diagrams were generated to display the number of genes able to be extracted using the poly(A)-enriched, AgSS (orange), the total RNA AgSS (yellow), or both (blue). The numbers in parentheses indicate the proportion of total protein-coding genes identified in each gene subset.


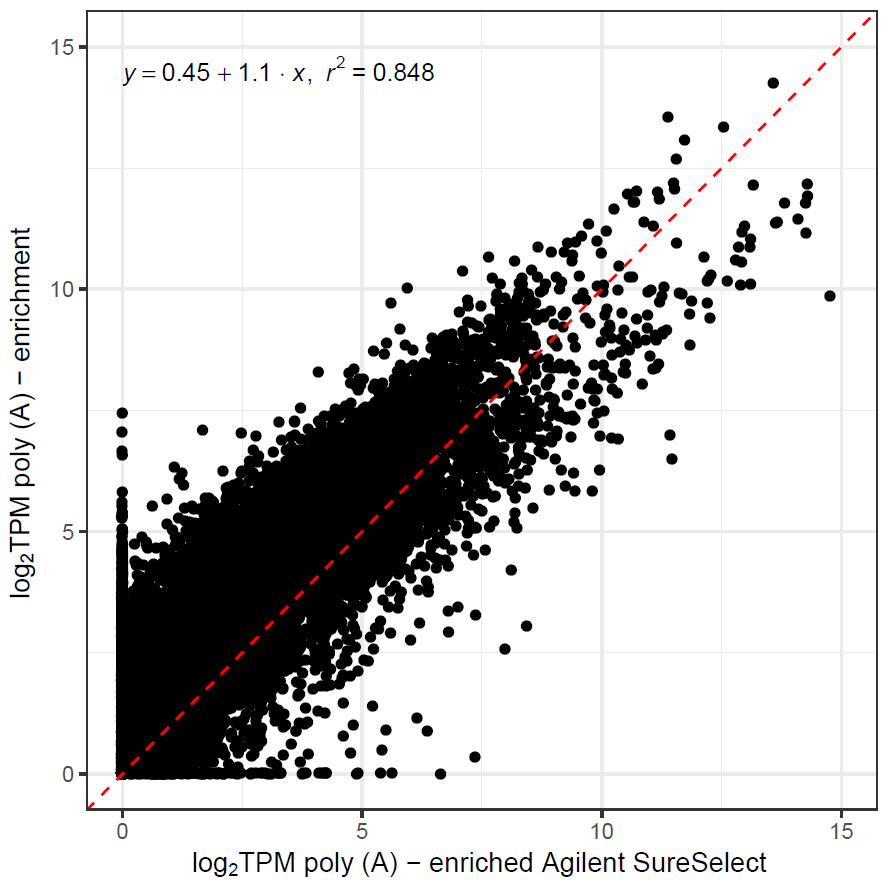


## Supplementary Figure 7. Comparison of the poly(A)-enrichment to the total RNA *A. fumigatus* AgSS library preparations of the transcriptomes of 2 dpi immunocompromised mouse

Using the sample taken at 2 dpi of an immunocompromised mouse, the log_2_ TPM values for mouse protein-coding genes in the poly(A)-enriched, *A. fumigatus* AgSS were plotted against the log_2_ TPM values for mouse protein-coding genes in the poly(A)-enrichment only. Genes with similar expression in both enrichments lie close to the identity line (x=y; red). Mouse genes whose expression values are more elevated in the poly(A)-enriched *A. fumigatus* AgSS sample compared to the poly(A)-enrichment sample lie below the identity line while genes more elevated in the poly(A)-enrichment compared to the poly(A)-enriched *A. fumigatus* AgSS lie above the identity line.

## Supplementary Dataset 1: RNA-Seq statistics for poly(A)-enriched AgSS and poly(A)-enrichment libraries from *B. malayi* samples collected following infection of the mosquito vector

## Supplementary Dataset 2: RNA-Seq statistics for AgSS and poly(A)-enrichment libraries from *A. fumigatus*

## Supplementary Dataset 3: RNA-Seq statistics for AgSS and rRNA-, poly(A)-depletion libraries from *w*Bm

## Supplementary Dataset 4: RNA-Seq statistics for *B. malayi* transcriptome data using AgSS with total RNA or poly(A)-enriched RNA
